# Range of Flexion Improvement in Degenerative Stages of the First Metatarsophalangeal Joint (*Hallux rigidus*) with Cross-Linked Hyaluronic Acid: A Cadaveric Study

**DOI:** 10.3390/jfmk9040259

**Published:** 2024-12-06

**Authors:** Annabel Capell Morera, Elena de Planell Mas, Laura Perez Palma, Maria Cristina Manzanares-Céspedes

**Affiliations:** 1 Clinical Sciences Department, Faculty of Medicine and Health Sciences, University of Barcelona, 08907 Hospitalet, Spain; annabelcapellmorera@ub.edu (A.C.M.); elenaplanell@ub.edu (E.d.P.M.); lperez@ub.edu (L.P.P.); 2 Human Anatomy and Embryology Unit, Experimental Pathology and Therapeutics Department, Faculty of Medicine and Health Sciences, University of Barcelona, 08907 Hospitalet, Spain

**Keywords:** hallux rigidus, viscosupplementation, anatomy, cadaveric study, articular goniometry, podiatry

## Abstract

Background: Viscosupplementation consists of intraarticular hyaluronic acid injections applied to treat pain and improve joint mobility. The objective of the study was to analyze the improvement of the range of mobility of the first metatarsophalangeal joint with a single dose of cross-linked hyaluronic acid. Methods: Ten fresh frozen specimens of feet sectioned below the knee were selected. Before and after the infiltration procedure, the range of flexion was calculated for all specimen’s metatarsophalangeal joints. To detect complications due to the procedure, five feet were dissected and five were sectioned with a diamond saw. Results: The range of the first metatarsophalangeal joint flexion differences between the preoperative and the postoperative period was as follows: (1) 47° (range, 37–51.5) to 58° (range, 49–69.5) degrees of loaded dorsiflexion (*p* > 0.006); (2) 41° (range, 40–51.5) to 58° (range, 52.5–66.5) degrees of unloaded dorsiflexion (*p* > 0.009); and (3) 14° (range, 10.5–24.25) to 16° (range, 14.25–28.5) degrees of unloaded plantarflexion (*p* > 0.083). No injuries of anatomical structures were observed either by anatomical dissection or in the anatomical sections. Conclusions: The results obtained in this viscosupplementation study demonstrate the improvement of the range of mobility of the first metatarsophalangeal joint without evidence of extravasation and lesions of the periarticular anatomical structures.

## 1. Introduction

Hallux rigidus (HR) is a progressive, degenerative osteoarthritis of the metatarsophalangeal joint of the first toe [1,2,3,4]. Clinically, it is characterized by pain, joint stiffness, a progressive loss of range of motion, mainly in dorsiflexion, and dorsal or periarticular osteophyte formation [4].

Different treatments for HR are described in the literature [5]. Treatment of HR may involve a variety of therapies and conservative or surgical interventions [6]. The conservative treatment of HR is used in initial/mild grades or in patients with medical or surgical situations that make it inadvisable. Conservative treatment includes pharmacological treatment (corticosteroid infiltrations and hyaluronic acid), physiotherapy (shock waves), custom orthotic insoles, and shoe modifications [7]. High-grade HR represents a complex disease characterized by several clinical and pathological findings, and to achieve optimal results, surgical treatment should be chosen among several surgical techniques depending on the degree of arthritis and other different clinical conditions [6]. Surgical techniques can be classified into two large groups: those that preserve the joint and those that sacrifice the joint. Examples of surgical techniques that preserve the joint are interposition arthroplasty, where joint movement is maintained by inserting a biological spacer into the joint [8], and cheilectomy [9]. Surgical techniques that sacrifice the joint are arthrodesis [10], arthroplasty [8], and Keller-type resection arthroplasty [11].

In recent years, viscosupplementation with hyaluronic acid (HA) has been the most widely used conservative treatment of HR [4,7,12,13,14,15]. Results from several clinical trials [4,12,13,14,15] demonstrate the effectiveness of intra-articular HA injections for pain relief, mentioning also anti-inflammatory and regenerative activity [13]. However, none of the articles report an improvement in the patient’s joint dynamics.

A new product (Desirial^®^ Plus, Vivacy, France) presented a novel formulation (cross-linked HA 21 mg/mL with mannitol), and the recent literature reported its efficacy in reducing pain and increasing collagen formation [16]. The objective is to test whether intra-articular treatment with cross-linked HA added with mannitol is feasible and if viscosupplementation increases the degrees of dorsiflexion and plantar flexion of the first MTP joint. A viscosupplementation procedure was simulated on unfixed cadaveric material. The manipulation of the product, the arthrocentesis, and the exploration of pre- and postoperative joint dynamics were recorded. Finally, in the cadaveric specimens, the distribution of the material and the eventual iatrogenic lesions in the different anatomical structures of the foot were analyzed.

## 2. Materials and Methods

Ten unpaired feet (five right and five left) were selected from fresh frozen non-bleeding Caucasian specimens. Specimens with scars related to previous surgical or traumatic procedures were excluded as well as the ones with foot deformities that could make the infiltration process difficult. Samples were included that showed bone thickening in the MTP joint (caused by dorsal osteophytes) and values between 10° and 60° of extension of the first MTP joint in the loading and unloading position, as assessed with a simple goniometer. This study was approved by the Bioethics Committee of the University of Barcelona (CBUB) on 12 June 2018.

The subjects were 4 men and 6 women with an average age of 87.5 years (range, 75.8–92.5). The selected below-knee samples presented various degrees of articular degeneration of the first MTP joint. The system used for the evaluation and selection of samples with HR is the classification of Coughlin and Shurnas [17]: two grade IV, six grade III, and two grade II (Table 1).

All specimens were submitted to an intra-articular infiltration of cross-linked HA 21 mg/mL with mannitol (Desirial^®^ Plus, Vivacy, France) performed in the medial part of the first MTP joint as an off-label use. The infiltration was performed by a podiatric surgeon with ten years of experience. The product was stained with methylene blue to analyze eventual extravasations. To compensate the density of the injection product, 27G needles (0.45 mm diameter × 12 mm long Sterican 27G hypodermic needle, BRAUN) were used.

**Figure 1:**
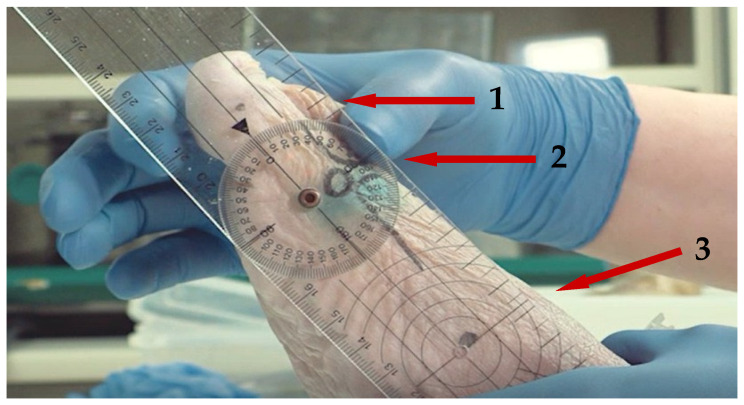
Reference points and position of the goniometer for the joint flexion assessment. (1) Interphalangeal joint of the hallux; (2) first MTP joint; (3) internal tuberosity of the navicular.

Before and after infiltration, the degrees of dorsiflexion and plantarflexion of the first MTP joint were measured in loading and unloading positions by articular goniometry. The assessment of the dorsiflexion of the first MTF joint was carried out with the specimen in an unloaded position, the same position in which the procedure is performed in the podiatric clinic with the patient seated. The evaluation of dorsiflexion in a loaded position was simulated by placing the sole of the foot of the specimen on the table. In both cases, the limb was held by the second operator. The dorsiflexion was applied manually by the first operator until joint resistance was encountered. The same procedure was then performed to assess the plantarflexion degrees of the first MTF joint in the unloaded position. This simulation of the clinical assessment of joint dynamics was carried out with a manual goniometer after marking the following reference points on the skin: the interphalangeal joint of the hallux, first MTP joint, and internal tuberosity of the navicular (Figure 1).

**Figure 2:**
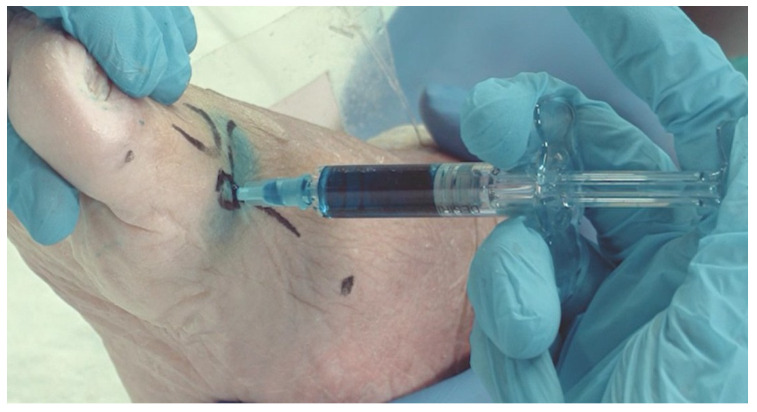
Technique and limits for intra-articular infiltration of cross-linked HA.

To perform the puncture for the intra-articular infiltration of cross-linked HA, the neurovascular and muscular anatomical structures must be taken into account. For this reason, the clock method described by Malagelada [18] is used to locate the safe injection zone, found between 11 o’clock and 1 o’clock of both feet. The injection area was detected by palpation and marked on the surgical area surface (Figure 1). Then, the viscosupplementation was carried out by injection of the stained product in the articular space via medial access (Figure 2) until resistance to the injection was evidenced. The volume of the product injected was recorded.

After having evaluated the degrees of movement of the MTP joint after infiltration, the samples were processed. The ten cadaveric samples used were randomly separated into two groups, the anatomical dissection group (five samples) and the sectional anatomy group (five samples).

In five samples, the dissection by planes of all the anatomical structures of the first metatarsal-digital segment was performed to see “in situ” the distribution of the HA in the first MTP joint, checking the correct execution of the application technique and that there was no deterioration of the adjacent anatomical structures [18].

The remaining five samples were subjected to a freezing process for at least six hours at a temperature between −17 °C and −20 °C to ensure the ideal conservation and section conditions. After the appropriate freezing time, sections of the first MTP joint were obtained from two samples in the sagittal plane and three samples in the transverse plane, using an Exakt 312 (Exakt, Nordernsted, Germany) diamond saw. Sagittal sections were obtained by following the first toe sagittal plane, while the transverse plane sections were obtained by following the coronal plane with the diamond saw. In both cases, parallel sections were performed to obtain 1 cm thick frozen blocks to maintain the original position and shape of the articular and periarticular anatomical structures. Both dissected specimens and sections were kept frozen after photography until the end of the study.

The dissection, carried out one week after the simulated surgeries, aimed to detect the eventual presence of lesions due to the injection procedure, both in the relevant anatomical structures of the first metatarsal-digital segment (first metatarsal, first MTP joint capsule, and phalanx of the proximal hallux) and in the skin layers, neurovascular anatomical structures, and muscles around the first metatarsal. The sectional observations, carried out in a unique section/photographical session one month after the simulated surgeries, permitted us to observe the distribution of the product into the articular cavity as well as the presence of eventual iatrogenic lesions or product extravasation. Two independent observers (a podiatrist (EPM) and an anatomist (MCM), each with 30 years of experience) made all assessments.

The variables collected are processed using the SPSS statistical package version 11.5.1. A description of the demographic and physical profile of the study subjects is made. Continuous variables are described using the statistical mean and interquartile range ([Q1; Q3]). Categorical variables are described as the number of cases and the percentage of the total. To determine whether there are differences between the degrees of plantar extension and flexion before and after the application of the HA, a comparative analysis is carried out using the Wilcoxon test for paired data. In addition, the effect size of the differences before and after the administration of the HA is evaluated using Cohen’s D coefficient.

## 3. Results

### 3.1. Range of Flexion of the First MTP Joint

The dorsiflexion degrees obtained under load before viscosupplementation reached a mean value of 47° (range, 22°–56°). Once the cross-linked HA was injected, the loaded dorsiflexion mean value was 58° (range, 42°–90°) (Table 2). The *p*-value of the Wilcoxon test for paired data was 0.006. Cohen’s d for statistically significant value was 1.730 (Table 3).

Significant differences were also found between degrees of unloaded flexion. A mean of 41 degrees of unloaded dorsiflexion (range, 24°–64°) was measured before the administration of cross-linked HA (Table 2). After injection, the statistical mean of the sample dorsiflexion measurements was 58° (range, 42°–72°). The *p*-value of the Wilcoxon test for paired data was 0.009, and Cohen’s d was 1.929 (Table 3).

The degrees of unloaded plantar flexion measured before HA administration obtained a statistical mean of 14° (range, 2°–40°). After viscosupplementation, the statistical mean measured was 16° (range, 6°–52°) (Table 2). The *p*-value of the Wilcoxon test for paired data was 0.083 and Cohen’s d was 0.450 (Table 3), thus not showing a statistically significant increase in the plantar flexion.

### 3.2. Plane-by-Plane Anatomical Dissection

**Figure 3:**
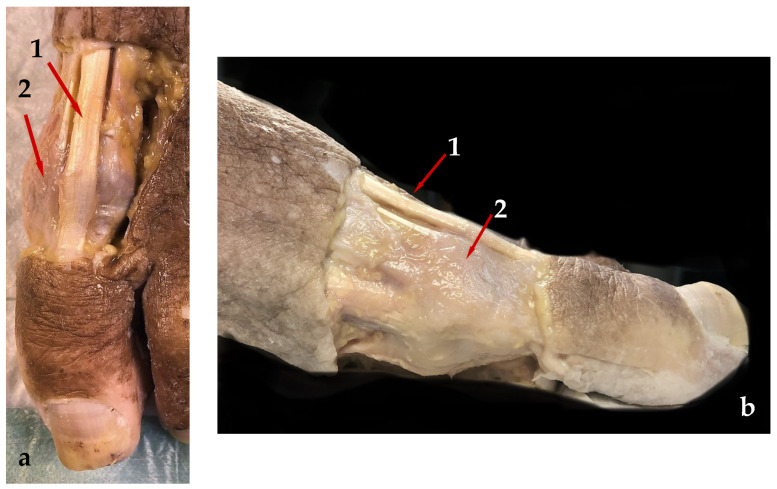
Plane-by-plane anatomical dissection results. (**a**). Dorsal view of a left foot, showing the unharmed EHL (1) and articular capsule (2) of the first metatarsophalangeal joint. (**b**). Lateral view of a left foot showing the EHL (1) and articular capsule (2) of the first metatarsophalangeal joint.

Plane-by-plane anatomical dissection of five randomly selected specimens revealed that in all cases, the colored cross-linked HA is visible within the joint capsule. None of the classically dissected samples showed evidence of extravasation of the product out of the articular space. In a similar manner, the exploration did not reveal lesions in the tissues or the extraarticular anatomical structures reported by Malagelada [18] to be susceptible to iatrogeny, including the Extensor hallucis longus (EHL) tendon and the articular capsule (Figure 3a,b).

### 3.3. Sectional Anatomy Analysis

**Figure 4:**
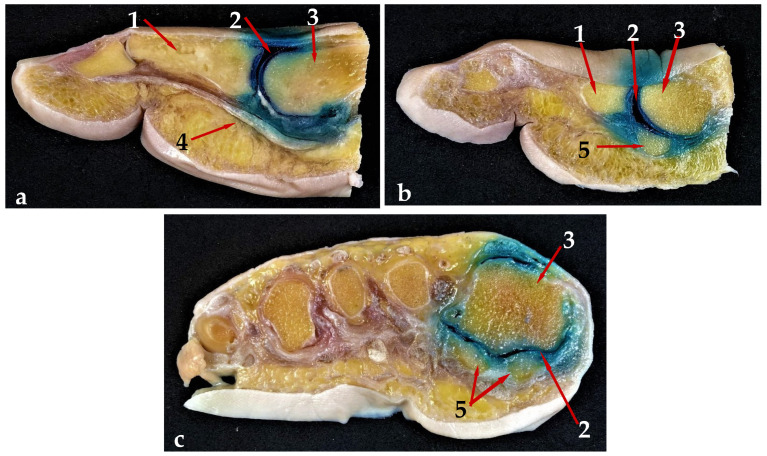
Sectional anatomy results. (**a**). Lateral view: section of the middle sagittal plane of the first toe, right foot. (1) proximal phalanx of the hallux; (2) first MTP joint space; (3) first metatarsal; (4) flexor hallucis longus; (**b**). Lateral view: section of the parasagittal plane of the first toe, right foot. (1) proximal phalanx of the hallux; (2) first MTP joint space; (3) first metatarsal; (5) lateral sesamoid bone. (**c**). Coronal view: left foot, axial cross section of the first metatarsal head (3), showing the inferior aspect of the first metatarsal joint space (2) at the level of the sesamoid bones (5).

Both the sagittal and the axial cross sections obtained showed that the colored HA was well contained within the intraarticular spaces of all the studied samples. Some of the methylene blue dye pervaded the adjacent anatomical structures during the intervention and immediately afterwards, as is visible in Figure 1 and Figure 2, but no evidence of product extravasation out of the articular space operated in was found when the samples were sectioned, and neither were any lesions found on the surrounding anatomic structures, including the flexor hallucis longus (FHL) and the sesamoid bones (Figure 4a–c).

## 4. Discussion

Despite the high prevalence of HR in the population over sixty years of age [1,19], there is very little scientific literature reporting its treatment by viscosupplementation with HA and, until recently, none about cross-linked HA. The present study presents the first results regarding the efficacy on cadaveric samples showing different degrees of articular degeneration of the first MTP joint of an arthrocentesis by applying a product (cross-linked HA with a concentration of 21 mg/mL with mannitol (Desirial^®^ Plus, Vivacy, France)) originally formulated for the treatment of dyspareunia in menopausal women as a potential (off-label) viscosupplementation material.

Endogenous HA is a component of synovial fluid that is essential for joint homeostasis [20]. In healthy joints, endogenous HA contributes to adequate lubrication, acts as a shock absorber for the transmission of loads across joint surfaces, and confers anti-inflammatory and antinociceptive properties of the synovial fluid [21,22]. In contrast, osteoarthritis causes a decrease in the concentration and molecular weight of endogenous hyaluronic acid, which reduces the mechanical and biological protection of the joint [22,23].

Exogenous HA is a substance produced from bacterial fermentation in vitro [23]. Exogenous HA preparations can be classified according to the molecular weight, whether a low molecular weight and a high molecular weight. The difference between these pharmaceutical preparations essentially lies in the size of the active molecule [24]. The local effects of infiltrations with HAs of different molecular weight in the intra-articular space have been reported by several studies. An experimental study performed by Sánchez Lazaro et al. [25] in 2010 using rabbits subjected to intra-articular injection in the knee, using two groups, each with a type of HA of the same molecular weight and different concentrations, concluded that the improvement of symptoms in living subjects is not influenced by the molecular weight of exogenous HA but by its concentration. They concluded that not all HA formulae with similar molecular weights are equivalent and that endogenous HA recovers its viscoelastic properties due to the improvement of the joint mechanical factors caused by the treatment. Iturriaga et al. [20] demonstrated in 2017 that a single intra-articular injection of low-molecular-weight HA into the osteoarthritic temporomandibular joint in rabbits resulted in better effects on articular tissues in the first thirty days, as compared to high-molecular-weight hyaluronic acid. However, after 135 days of infiltration, both groups showed a regression of joint tissue repair.

A high-molecular-weight and high-concentration cross-linked HA was used based on the following arguments:

1. Cohesiveness, understood as the ability of a material to maintain its stability due to the interaction of internal forces, is high. High-molecular-weight HA cannot pass through the pores of the cell wall. This prevents the HA from diffusing through adjacent structures during joint movement. With low-molecular-weight HA, the opposite occurs; it diffuses more easily through the effect of osmosis [26].
2. The chemical structure of high-molecular-weight HA is a network of polymer chains intertwined with each other, forming a very large molecule, allowing it to remain localized on the injection surface, without penetrating deeper layers of the different tissues, thus providing more volume. Low-molecular-weight HA, on the other hand, is a smaller molecule with a very finely chopped linear polymer chain to facilitate its absorption and thus reach deeper layers of the dermis [27].
3. An HA formula that lasts over time is needed. Enzymatic degradation depends on molecular weight. The union of intertwined polymer chains that form a mesh that high-molecular-weight HA also presents then protects it from the enzymes of the internal environment and gives it a longer shelf life, contrary to what happens with low-molecular-weight HA, which degrades rapidly in contact with enzymes [27].
4. The anti-inflammatory and immunosuppressive properties of high-molecular-weight HA molecules are better in contrast to the pro-inflammatory properties of low-molecular-weight molecules [27,28].
5. Mannitol is a natural antioxidant molecule that acts by eliminating the most aggressive hydroxyl radicals generated during infiltration, minimizing the degradation of HA, and acting as a thermal stabilizer, guaranteeing stability and physical-chemical properties throughout the product’s shelf life [29].

HA infiltrations have been reported as a minimally invasive, pharmacologically based treatment to decrease pain and reduce inflammation with fewer undesirable effects and more beneficial effects than other arthrocentesis products [1]. Currently, HA is used to heal infected ulcers in diabetic patients [30,31], Morton’s Neuroma [32], the ankle joint [33], and metatarsophalangeal arthritis [4], among others. Recent clinical trials have been able to demonstrate that HA shows a statistically high effectiveness to treat arthritic processes, specifically in synovial joints such as knee, hip, and lumbar joints [22,23,34,35,36]. However, the treatments reported, involving the application of non-cross-linked HA at low concentrations, were not homogeneous.

The results of the five clinical trials conducted so far for HR support the efficacy of HA injections in relieving pain and improving joint function. Four of these studies analyze the results of non-cross-linked HA at different concentrations. Two non-comparative studies analyzed only the use of non-cross-linked HA [13,15]; one study compared non-cross-linked HA with a placebo group (saline) [14]; and a third study compared the results between non-cross-linked HA and triamcinolone acetonide [4]. Finally, a single study analyzed the clinical effectiveness of HA cross-linked with mannitol [12] in the first MTP joint.

The type of cross-linked HA used was originally applied in gynecological protocols. By means of a single injection, it helps to reconstitute moderately or severely hypertrophied tissue in the genital area and, above all, causes a lasting increase in the volume of the labia majora, improving the inflammatory symptoms of patients [37]. The criterion proposed in the present study is to analyze the feasibility of injecting this off-label product in viscosupplementation procedures without causing damage to the surrounding anatomical structures in order to improve the range of motion of the affected joint. So far, only two studies have described the application of this product in the first MTP joint [12,38] in patients, reporting moderate postoperative pain lasting no more than 6 h after injection as the only adverse effect.

The main strength of this study is that it constitutes a surgical proof of concept that first evaluates product handling in an arthrocentesis procedure and then eventual complications, such as punctures or extravasations, using cadaveric specimens. The anatomic specimens selected met the inclusion criteria related to the clinical or anatomical severity of the pathology, constituting a study population as close as possible to the daily clinical practice patients. This study also allows the evaluation of the first MTP joint range of movement in selected specimens showing different stages of arthritic degeneration. Afterwards, the samples were either sectioned with a diamond saw or dissected by planes as previously described to re-check the safety of the procedure in terms of extravasations and/or punctures or lesions to the periarticular anatomical structures in order to prevent eventual complications in routine clinical practice [18].

The results obtained indicate an increase in the degrees of flexion measured in joints with severe and moderate degenerative HR, while the less severe cases showed discrete increases in the range of movement. The fact that the applied product is a denser cross-linked HA supports the initial hypothesis that it would provide an improvement of the joint range of movement even if injected in small quantities. In the recent HR viscosupplementation studies conducted by Pons et al. [4], Galois et al. [12], Maher et al. [13], Munteanu et al. [14], and Petrella et al. [15], no mention is made about an improvement of the first MTP joint range of movement, since most report only the pain reduction experienced by the patients.

## 5. Conclusions

The intra-articular injection of 21 mg/mL of HA cross linked with mannitol in cadaveric samples of patients affected by different degrees of degeneration of the first MTP joint produces a notable improvement in joint mobility, statistically significant in dorsiflexion but not in plantarflexion, providing no evidence of a significant risk of causing damage to the adjacent anatomical structures. Further studies will be needed to demonstrate if this increased range of motion is related to changes in the articular volume.

Moreover, future studies should have a larger sample size as this would allow for better extrapolation of the results to clinical practice. This type of treatment with patients who present a clinical picture similar to HR or with groups of patients with different degrees of joint degeneration could contribute to improving their gait cycle, as well as obtaining postural improvements and, by extension, also improving their quality of life.

## Figures and Tables

**Table 1:** Demographic data and sample physical characteristics.

Total Sample Size: n = 10	
Sex, n (%)	
Female	6 (60%)
Male	4 (40%)
Age in years, mean [Q1; Q3]	87.5 [75.8; 92.5]
Degree of arthrosis involvement, n (%):	
High (grade IV)	2 (20.0%)
Moderate–High (grade III)	6 (60.0%)
Moderate (grade II)	2 (20.0%)

**Table 2:** Results in degrees of flexion of the first MTP joint of the cadaveric parts before and after infiltration. H = high; M-H = moderate–high; M = moderate.

Sample Number	Sex	Degree of Joint Involvement	Before Injection Hyaluronic Acid	After Injection Hyaluronic Acid	Before Injection Hyaluronic Acid	After Injection Hyaluronic Acid	Before Injection Hyaluronic Acid	After injection Hyaluronic Acid
			Degrees of Unloaded Plantarflexion	Degrees of Unloaded Plantarflexion	Degrees of Loaded Dorsiflexion	Degrees of Loaded Dorsiflexion	Degrees of Unloaded Dorsiflexion	Degrees of Unloaded Dorsiflexion
1	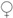	M-H	12	15	36	42	50	68
2	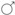	M-H	25	36	32	48	40	60
3	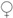	M-H	10	16	50	90	64	72
4	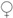	M-H	22	30	40	52	40	50
5	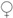	M-H	40	52	50	68	52	70
6	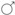	H	16	34	56	70	24	52
7	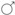	M-H	2	6	44	58	40	56
8	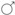	M	8	10	52	58	42	54
9	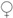	H	16	24	22	46	24	42
10	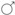	M	12	14	54	76	62	62

**Table 3:** Results: means and *p*-values of the first MTP joint of the cadaveric samples before and after infiltration. * statistically significant; # non statistically significant.

	n	Before Infiltration		After Infiltration		
		Means	[Q1; Q3]	Means	[Q1; Q3]	*p*-Value
Degrees of loaded dorsiflexion	10	47	[22–56]	58	[42–90]	0.006 *
Degrees of unloaded dorsiflexion	10	41	[24–64]	58	[42–72]	0.009 *
Degrees of unloaded plantarflexion	10	14	[2–40]	16	[6–52]	0.083 #

## Data Availability

Data collected on samples from voluntary donors are already provided in the article. Other images and data cannot be made available due to restrictions imposed by the ethics approval obtained for the present study.
